# Effects of treatment with corticosteroids on human rhinovirus-induced asthma exacerbations in pediatric inpatients: a prospective observational study

**DOI:** 10.1186/s12890-023-02798-6

**Published:** 2023-12-05

**Authors:** Keiko Kan-o, Yasuyoshi Washio, Takeshi Oki, Tsuguto Fujimoto, Takahito Ninomiya, Makoto Yoshida, Masaki Fujita, Yoichi Nakanishi, Koichiro Matsumoto

**Affiliations:** 1 Department of Respiratory Medicine, Graduate School of Medical Sciences, Kyushu University, 3-1-1 Maidashi, Higashi-ku, Fukuoka, 812-8582 Japan; 2 Division of Pediatrics, National Hospital Organization Fukuoka National Hospital, Fukuoka, Japan; 3 Department of Fungal Infection, National Institute of Infectious Diseases, Tokyo, Japan; 4 Division of Respiratory Medicine, National Hospital Organization Fukuoka National Hospital, Fukuoka, Japan; 5 Department of Respiratory Medicine, Fukuoka University School of Medicine, Fukuoka, Japan; 6 Division of Respirology, Department of Medicine, Fukuoka Dental College, Fukuoka, Japan

**Keywords:** Pediatric asthma, Corticosteroids, Exacerbation, Human rhinovirus

## Abstract

**Background:**

Human rhinoviruses (HRVs) infection is a common cause of exacerbations in pediatric patients with asthma. However, the effects of corticosteroids on HRV-induced exacerbations in pediatric asthma are unknown. We conducted a prospective observational study to determine the viral pathogens in school-age pediatric inpatients with asthma exacerbations. We assessed the effects of maintenance inhaled corticosteroids (ICS) on the detection rates of HRV species and treatment periods of systemic corticosteroids during exacerbations on pulmonary lung function after exacerbations.

**Methods:**

Nasopharyngeal samples and clinical information were collected from 59 patients with asthma exacerbations between April 2018 and March 2020. Pulmonary function tests were carried out 3 months after exacerbations in 18 HRV-positive patients. Changes in forced expiratory volume in 1 second (FEV_1_)% predicted from baseline in a stable state were compared according to the treatment periods of systemic corticosteroids.

**Results:**

Fifty-four samples collected from hospitalized patients were analyzed, and viral pathogens were identified in 45 patients (83.3%) using multiplex PCR assay. HRV-A, −B, and -C were detected in 16 (29.6%), one (1.9%), and 16 (29.6%) patients, respectively. The detection rates of HRV-C were lower in the ICS-treated group compared with those in the ICS-untreated group (*p* = 0.01), whereas maintenance ICS treatment did not affect the detection rate for viral pathogens in total and HRV-A. Changes in FEV_1_% predicted in patients treated with systemic corticosteroids for 6–8 days (*n* = 10; median, 4.90%) were higher than those in patients treated for 3–5 days (*n* = 8; median, − 10.25%) (*p* = 0.0085).

**Conclusions:**

Maintenance ICS reduced the detection rates of HRV (mainly HRV-C) in school-age inpatients with asthma exacerbations, and the treatment periods of systemic corticosteroids during exacerbations affected lung function after HRV-induced exacerbations. The protective effects of corticosteroids on virus-induced asthma exacerbations may be dependent upon the types of viral pathogen.

**Supplementary Information:**

The online version contains supplementary material available at 10.1186/s12890-023-02798-6.

## Background

A viral infection in the respiratory tract can exacerbate asthma and cause unscheduled visits to physicians and hospital admissions. Severe exacerbations of asthma are associated with a decline in lung function in children and adults [1]. Respiratory viruses have been detected in ≤80% of children with asthma exacerbations using reverse transcriptase-polymerase chain reaction (RT-PCR).

Human rhinoviruses (HRVs) are the most frequently identified viruses during exacerbations [2–4]. More than 160 strains of HRVs have been identified. They have been classified into three genetically distinct species: HRV-A, HRV-B, and HRV-C [5]. Infection with HRV-A or HRV-C has been associated with hospitalization for acute respiratory illness, whereas HRV-B infection tends to cause fewer symptoms [6, 7]. Recent clinical studies have shown that HRV-C (the species identified most recently) may be associated with increased disease severity and asthma risk in young children [8–10].

Based on studies in preschool and school-age children, inhaled corticosteroids (ICS) are the most efficacious in improving asthma control and exacerbation prevention in children with evidence of type 2 inflammation [11, 12]. A randomized clinical trial by Fitzpatrick et al. showed that a phenotype of children with type 2 inflammation evidenced by aeroallergen sensitization and increased blood eosinophil counts for whom daily ICS treatment conferred the most protection against symptoms and exacerbations [11]. Conversely, in vitro and in vivo studies have suggested that corticosteroids (e.g, budesonide and fluticasone propionate) may impair innate immune responses to viruses and therefore prolong viral infection [13, 14]. Whether maintenance ICS have protective or detrimental effects on virus-induced exacerbations in children with asthma is not known. Systemic corticosteroids, potent anti-inflammatory agents, are recommended for severe exacerbations of asthma and are usually prescribed for 3 days or 5 days in children because of concerns over adrenal suppression, growth inhibition, and bone disease [15–17]. However, a recent Cochrane Database review showed no clear evidence of the appropriate way of using systemic corticosteroids during exacerbations in children with asthma [18]. Moreover, whether the use of systemic corticosteroids during exacerbations affects pulmonary function after exacerbations is not known.

We conducted a single-center prospective observational study to detect viral pathogens in hospitalized children with asthma exacerbations and evaluated the effects of maintenance ICS for the detection of HRV strains. We also assessed whether the treatment period of systemic corticosteroids during HRV-induced exacerbations affects pulmonary lung function 3 months after exacerbations.

## Methods

### Patient enrollment and treatment

This prospective observational study recruited pediatric patients aged 6–16 years with asthma exacerbations who were hospitalized in the Fukuoka National Hospital (part of the National Hospital Organization) between April 2018 and March 2020.

A physician-based diagnosis of asthma and exacerbations and the criteria for using systemic corticosteroids for exacerbations were made according to the Japanese Pediatric Guideline for the Treatment and Management of Asthma [19]. Briefly, the diagnosis of asthma was made comprehensively, referring to the allergic predisposition, clinical symptoms such as recurrent paroxysmal wheezing and dyspnea, examination findings, and laboratory findings such as reversible airflow limitation and increased airway hyperresponsiveness. In addition, diseases other than asthma presenting with similar symptoms were excluded. Prednisolone sodium succinate (1 mg/kg of body weight, 3 or 4 times a day) was administered intravenously to all patients treated with systemic corticosteroids. Treatment with prednisolone sodium succinate was discontinued after the physician confirmed the disappearance of wheezes and improvement in desaturation or lung function.

### Collection of samples and data

**Fig. 1:**
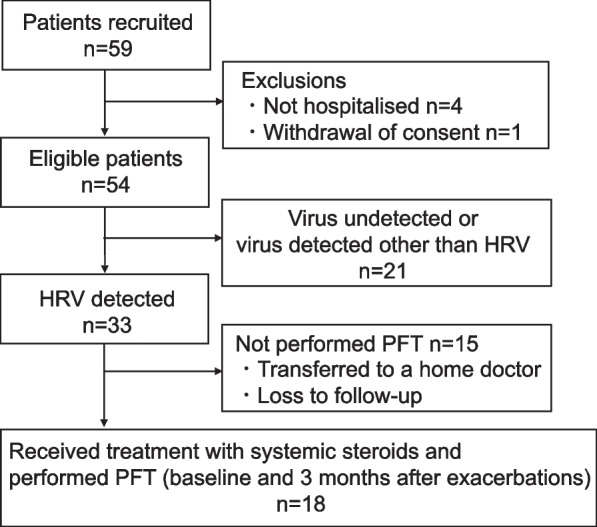
The flow chart of this study. HRV, human rhinovirus; PFT, pulmonary function test

Nasopharyngeal swabs were taken and suspended in a universal transport medium (Copan, Brescia, Italy). Samples were stored immediately at − 80 °C and transported every month to the Department of Respiratory Medicine within Fukuoka University School of Medicine for virus detection. The information and laboratory data of patients were obtained from medical records and collected via a system for capturing electronic data. Data from pulmonary function tests (PFTs; measured when the child had a stable status) from 1 year before to 7 days before hospitalization for exacerbations were collected from some HRV-positive patients as a baseline. PFTs were carried out again 3 months after exacerbations. A flow chart of this study is shown in Fig. 1.

### Multiplex real-time PCR

Viruses were detected as described previously [20, 21]. Nucleic acids were extracted from 200 μL of the viral transport medium (Qiagen, Stanford, VA, USA) according to manufacturer instructions. Ten microliters of extracted nucleic acids were tested using xTAG-RVP (Luminex, Austin, TX, USA) and analyzed on a MAGPX® system (Luminex) per manufacturer protocols. Luminex xTAG-RVP assays can be used to detect nucleic acids from 19 viruses that cause infections in the upper respiratory tract, including adenoviruses, coronaviruses (229E, HKU1, OC43, NL63), human metapneumovirus, HRV/human enteroviruses, influenza A virus (H1/2009, H1 and H3), influenza B virus, parainfluenza virus types 1–4, respiratory syncytial virus, and human bocaviruses.

### HRV typing

RNA from nasal samples positive for HRVs/human enteroviruses according to the Luminex xTAG-RVP assay was transported to the National Institute of Infectious Diseases (Tokyo, Japan) for HRV typing and detection of human enterovirus D68, as described previously with slight modification [22]. Briefly, viruses were detected by nested reverse transcription-PCR using primers targeting consensus VP4/VP2 coding regions and their genogroups identified by sequencing. The first PCR primer set was P-2 (5′-CCTCCGGCCCCTGAATGCGGCTAAT-3′) and E31 (5′-TCTGGTAACTTCCACCACCA-3′). The second PCR primer set was EVP4 (5′-CTACTTTGGGTGTCCGTGTT-3′) and OL68–1 (5′-GGTAAYTTCCACCACCANCC-3′).

### Statistical analyses

Statistical analyses were undertaken using R 4.0.3 (R Institute for Statistical Computing, Vienna, Austria). Continuous variables are shown as the mean and standard deviation or median and interquartile range using the Student’s *t*-test or nonparametric Mann–Whitney *U*-test. Categorical variables are presented as numbers and percentages. The chi-square test or Fisher’s exact test was used for categorical variables, as appropriate. Data on pulmonary function were analyzed using Prism 9 (GraphPad, San Diego, CA, USA). The nonparametric Mann–Whitney *U*-test was employed to compare two sets of data, and values are expressed as the median with an interquartile range. *p* < 0.05 (two-sided) was considered significant.

## Results

### Participant characteristics, laboratory findings, and clinical outcomes

Fifty-nine school-age inpatients with asthma exacerbations were recruited prospectively between April 2018 and March 2020, and 54 patients were eligible for analysis (Fig. 1). Participants were predominantly male (57.4%) and had a mean age of 9.8 (SD, 2.4) years (Table 1). For maintenance therapy, 28 participants (51.8%) received any ICS, and 22 (40.7%) received ICS plus a long-acting beta_2_ agonist. No patients received daily systemic corticosteroids for maintenance therapy before exacerbations, and one patient was treated with anti-immunoglobulin (Ig) E antibody. Most participants (85.2%) had symptoms of wheezing at exacerbations, and 53 (98.1%) received systemic corticosteroids during exacerbations (for a median duration of 5 days).

**Table 1:** Characteristics, laboratory findings, and clinical outcomes of participants

	All *n* = 54
Age, years^a^	9.8 ± 2.4
Male, n (%)	31 (57.4%)
Treatment during a stable state, n (%)	
ICS	6 (11.1%)
ICS/LABA	22 (40.7%)
Anti-lgE antibody	1 (1.9%)
Symptom	
Wheezes, n (%)	46 (85.2%)
Duration of URTI symptoms before consultation, days^b^	3 (2–6)
Body temperature, °C^a^	37.3 ± 0.8
Laboratory findings	
CRP, mg/dL^b^	0.49 (0.11–1.15)
WBC count, /μL^a^	8453 ± 2763
Eosinophil count in bloods, /μL^b^	130 (11–537)
Eosinophil percentage in blood, %^b^	1.5 (0.1–6.3)
Total serum IgE, IU/mL^b^	1050 (595–1891)
Hospitalization duration, days^b^	7 (6–8)
Treatment with systemic corticosteroids, n (%)	53 (98.1%)
Duration with systemic corticosteroids, days^b^	5 (4–7)

Data are the mean ± standard deviation^a^ and the median (interquartile range)^b^

Number of participants with missing data were as follow: 2 for CRP; 1 for WBC count and eosinophil count and percentage in blood, 8 for total serum IgE

*ICS* inhaled corticosteroids, *LABA* long-acting beta_2_-agonist, *URTI* upper respiratory tract infection, *CRP* C-reactive protein, *WBC* white blood cell, *IgE* immunoglobulin E

### Detection of viral pathogens

Viral pathogens were detected in nasopharyngeal swabs from 45 of 54 pediatric patients with asthma exacerbations (83.3%) (Table 2). The most commonly identified pathogens by multiplex PCR assay were HRVs/enteroviruses (66.7%), parainfluenza viruses (9.3%), and respiratory syncytial virus (5.6%). Of 36 HRV/enterovirus-positive samples, 33 were HRV-typed. HRV-A, HRV-B, and HRV-C were detected in 16 (29.6%), one (1.9%), and 16 (29.6%) samples, respectively. Enterovirus D68 was detected in one (1.9%) sample. More than one type of viral pathogen was detected in five patients (9.3%).

**Table 2:** Detection of specific viral pathogen

	All (*n* = 54)
	*n* (%)
Viral pathogen detected	45 (83.3%)
HRV/enterovirus^a^	36 (66.7%)
HRV	33 (61.1%)
HRV-A	16 (29.6%)
HRV-B	1 (1.9%)
HRV-C	16 (29.6%)
Enterovirus D68	1 (1.9%)
Non-detected	2 (3.7%)
Parainfluenza virus	5 (9.3%)
Parainfluenza type 1 virus	1 (1.9)
Parainfluenza type 3 virus	2 (3.7)
Parainfluenza type 4 virus	2 (3.7)
Respiratory syncytial virus	3 (5.6%)
Influenza A virus	2 (3.7%)
Influenza A virus 2009 H1N1	2 (3.7%)
Human metapneumovirus	1 (1.9%)
Coronavirus	1 (1.9%)
Coronavirus OC43	1 (1.9%)
Adenovirus	1 (1.9%)
Human bocavirus	1 (1.9%)
Two types of virus detected	5 (9.3%)
HRV-A + parainfluenza virus	2 (3.7%)
HRV-A + human bocavirus	1 (1.9%)
HRV-C + respiratory syncytial virus	1 (1.9%)
Coronavirus + respiratory syncytial virus	1 (1.9%)

Data were presented as the number (percentage)

*HRV* human rhinovirus

^a^Detected by multiplex PCR assay

### Effects of maintenance ICS treatment upon HRV detection

**Fig. 2:**
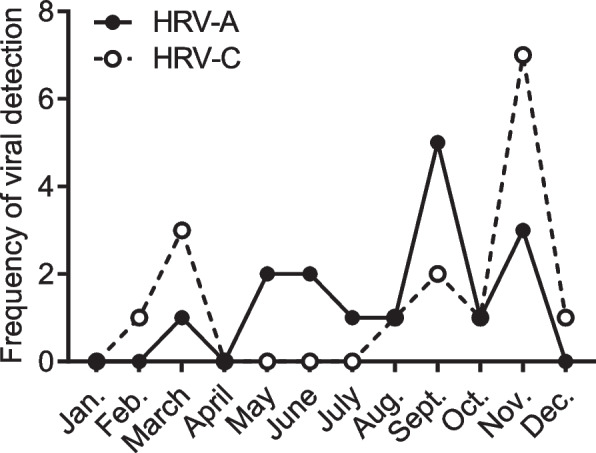
Frequency of detection of HRV-A or HRV-C in hospitalized school-age children with asthma exacerbations. HRV, human rhinovirus

There was no significant difference in the detection rates of viral pathogens in total between patients treated (78.6%) or not treated with ICS (88.5%) (Table 3). However, the detection rates of HRVs (80.8% in the non-ICS group vs. 42.9% in the ICS group), especially HRV-C (46.2% in the non-ICS group vs. 14.3% in the ICS group), were lower in the ICS group than in the non-ICS group, whereas ICS treatment did not affect the detection rate for HRV-A. Patients treated with ICS showed lower WBC counts compared with patients untreated with ICS, whereas there were no differences in other laboratory findings, clinical outcomes, or the detection rates of viral pathogens except for HRV (Additional file 1). Figure 2 shows the detection of HRV-A and -C each month. HRV-A was detected almost throughout the entire season, whereas HRV-C showed more season-based detection (Fig. 2).

**Table 3:** Comparison of HRV detection between patients with or without ICS treatment

	All (*n* = 54)	Non-ICS group (*n* = 26)	ICS group (*n* = 28)	*p*-value
	*n* (%)	*n* (%)	*n* (%)	
Viral pathogen detected	45 (83.3%)	23 (88.5%)	22 (78.6%)	0.47
HRV	33 (61.1%)	21 (80.8%)	12 (42.9%)	0.004*
HRV-A	16 (29.6%)	9 (34.6%)	7 (25.0%)	0.44
HRV-B	1 (1.9%)	0 (0.0%)	1 (3.6%)	1.00
HRV-C	16 (29.6%)	12 (46.2%)	4 (14.3%)	0.01*

*ICS* inhaled corticosteroids, *HRV* human rhinovirus

*p < 0.05 by Chi-squared test or Fisher’s exact test between Non-ICS group and ICS group

### Effects of treatment periods of systemic corticosteroids on pulmonary function after HRV-induced exacerbations

**Fig. 3:**
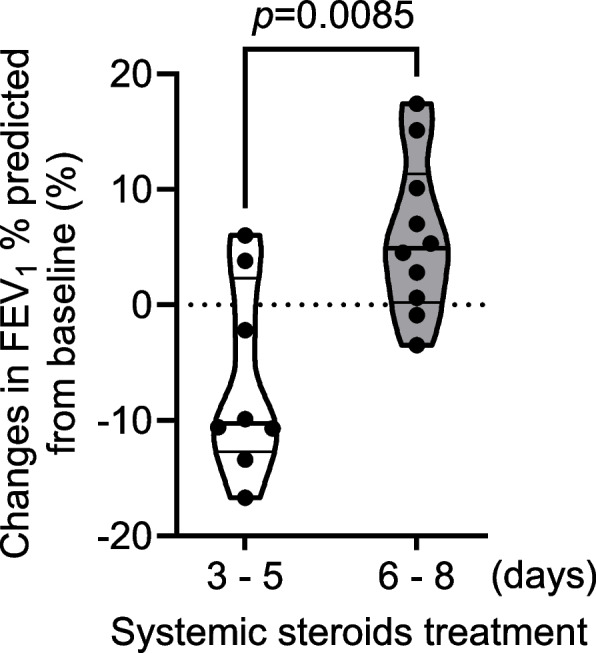
Comparison of changes in FEV_1_% predicted from baseline in HRV-positive patients. Eighteen HRV-positive patients underwent a PFT 3 months after exacerbations, and FEV_1_% predicted from baseline was compared with the treatment period of systemic corticosteroids (3–5 days, *n* = 8; 6–8 days, *n* = 10). Values are expressed as the median with an interquartile range. Differences in data were analyzed by the Mann–Whitney *U*-test. FEV_1_, forced expiratory volume in 1 second

Finally, we investigated whether the treatment periods of systemic corticosteroids during exacerbations affected pulmonary function 3 months after an exacerbation. Of 33 HRV-positive patients, 18 underwent a PFT (11 HRV-A, one HRV-B, and 6 HRV-C), and all needed to be treated with systemic corticosteroids. Compared with patients who did not undergo a PFT, patients who underwent a PFT had a higher likelihood of having ICS treatment, whereas differences in other baseline characteristics, laboratory findings, or clinical outcomes were not observed (Additional file 2). Changes in forced expiratory volume in 1 second (FEV_1_)% predicted from baseline in a stable state were compared according to the treatment periods of systemic corticosteroids. Changes in FEV_1_% predicted in patients treated with systemic corticosteroids for 6–8 days (*n* = 10; median, 4.90%) were higher than those in patients treated for 3–5 days (*n* = 8; median, − 10.25%) (Fig. 3). Multiple viruses were detected in 3 patients treated with systemic corticosteroids for 6–8 days. Two were detected with HRV-A and parainfluenza virus, and one with HRV-C and respiratory syncytial virus. There were no differences in characteristics at baseline or laboratory findings between patients treated with systemic corticosteroids for 3–5 days or 6–8 days (Additional file 3). No changes in FEV_1_% were predicted between HRV-A-positive and HRV-C-positive patients (Additional file 4).

## Discussion

Similar to previous reports, viral pathogens were detected in ~ 80% of asthma exacerbations in hospitalized school-age children in the present study. HRV-A (29.6%) and HRV-C (29.6%) were predominantly detected viruses. Virus-induced exacerbation could occur with a similar prevalence despite ICS maintenance therapy, but ICS reduced the detection rates of HRV (especially HRV-C). Patients whose asthma was exacerbated by HRVs and treated with systemic corticosteroids for 3–5 days did not recover their lung function to that observed at baseline, and extended treatment (6–8 days) with systemic corticosteroids improved lung function 3 months after exacerbations. These data suggested that systemic corticosteroids treatment for ≤5 days was insufficient to prevent prolonged airway inflammation.

Studies have shown that even low-dose ICS can reduce exacerbations and the risk of death from asthma [23, 24]. However, to the best of our knowledge, no studies have ascertained if ICS reduce or increase the rate at which respiratory viral infections trigger a sequence of inflammatory events that result in an exacerbation. Neil et al. reported that school-age children who presented to the emergency department with worsening asthma were significantly less likely to have reported use of ICS and were more likely to have a respiratory virus compared with a case–control group of children with similar asthma severity [25]. In our study, ICS treatment did not affect the total detection rate of viral pathogens, but ICS reduced the detection rates of HRV-C in hospitalized school-age children with asthma exacerbations. These results suggest that ICS may specifically suppress an HRV-C-induced excessive immune response, thereby preventing exacerbations. HRV-C (a newly identified HRV species) is associated with recurrent wheezing in infants, acute asthma severity in children, and an increased prevalence of hospital admissions for acute-wheezing exacerbations in young children [8–10]. Intercellular adhesion molecule-1 is a cellular receptor for most HRV-A and all HRV-B molecules. In contrast, HRV-C binds cadherin-related family member 3 (CDHR3) to gain entry into host cells [26]. A single-nucleotide polymorphism in the *CDHR3* rs6967330 locus has been linked to severe exacerbations of asthma and increased susceptibility to HRV-C infections in young children, possibly because of increased HRV-C binding and viral replication by the *CDHR3* polymorphism [27–29]. One clinical trial showed that experimental infection with HRV-16 (an HRV-A species) in asthma patients increased intercellular adhesion molecule-1 expression in the bronchial epithelium regardless of ICS treatment [30]. An in vitro study also reported that budesonide did not reduce replication of HRV-16 in bronchial epithelial cells from healthy people or patients with asthma [31]. These findings suggest that maintenance ICS may not prevent HRV-A infection and subsequent viral replication in asthmatic patients. However, in vitro studies focusing on HRV-C infection have been scarce, and the effects of ICS on CDHR3 expression have been unclear because HRV-C can replicate only in ciliated epithelial cells [32]. Further studies are needed to elucidate if treatment with ICS is protective for HRV-C-induced exacerbations in children with asthma.

Severe exacerbations of asthma in children are associated with a rapid decline in lung function [1]. Nevertheless, whether the use of systemic corticosteroids during exacerbations affects pulmonary function after asthma exacerbations is not known. Only one clinical trial has assessed the effects of different doses of systemic corticosteroids during exacerbations on pulmonary function upon discharge in hospitalized children with asthma exacerbations, and the results were inconsistent [33]. Even in adult patients with asthma, studies have not identified a conclusive benefit of one regimen of systemic corticosteroids upon lung function over another regimen during exacerbations [34, 35]. Those studies did not consider the pathogenesis of asthma exacerbations, which may be why clinical evidence is lacking. In the current study, all patients who had a PFT had HRV-induced severe asthma exacerbations. In virus-induced severe exacerbations, administration of systemic corticosteroids for 3–5 days may not be sufficient to control excessive inflammation and may result in a prolonged decline in lung function; more extended use of systemic corticosteroids may ameliorate this effect.

This study detected more than one type of viral pathogen in five patients (9.3%). Co-detection with multiple viruses is consistent with previous studies of respiratory pathogens in children with asthma exacerbations [36–38]. Some studies have shown an association between co-infection and clinical severity of exacerbations [36, 37]. In this study, there were no differences in characteristics and laboratory findings or duration of hospitalization and treatment with systemic corticosteroids between patients with a single virus detected (*n* = 40) or multiple viruses detected (*n* = 5) (data not shown). The significance of co-detections and virus–virus interactions have not been fully elucidated. Further studies using comprehensive testing are needed to determine their impact.

Our study had limitations. The sample size was relatively small, and approximately half of HRV-positive children had a PFT. We did not collect data on changes in maintenance therapy before and after exacerbations, which may influence pulmonary lung function. Also, we did not collect data on the side effects of systemic corticosteroids. However, a systematic review of 85 trials in children with acute respiratory disorders concluded that use of the systemic corticosteroids for ≤14 days was not associated with an increase in the prevalence of adverse events across organ systems [39].

## Conclusions

HRV-A and HRV-C are associated mainly with asthma exacerbations in hospitalized school-age children. Maintenance ICS reduces the detection rates of HRV-C but not HRV-A. The treatment periods of systemic corticosteroids during exacerbations affect lung function after HRV-induced exacerbations**.** The present study suggests that the protective effects of corticosteroids on virus-induced asthma exacerbations may be dependent upon underlying species-specific factors.

### Supplementary Information

**Additional file 1.**

**Additional file 2.**

**Additional file 3.**

**Additional file 4.**

## Data Availability

The datasets generated and/or analyzed during the current study are available from the corresponding author on reasonable request.

## Abbreviations

HRV: Human rhinovirus
ICS: Inhaled corticosteroids
PFT: Pulmonary function test
FEV_1_: Forced expiratory volume in one second
CDHR3: Cadherin-related family member 3
