# Supplementary material for: Effects of treatment with corticosteroids on human rhinovirus-induced asthma exacerbations in pediatric inpatients: a prospective observational study
Source: BMC Pulm Med. 2023 Dec 5;23:487. doi: 10.1186/s12890-023-02798-6 (PMC10696820; doi:10.1186/s12890-023-02798-6)
Supplement: Supplementary file 1 — Additional file 1. [file 12890_2023_2798_MOESM1_ESM.pdf]

Additional file 1. Comparison of characteristics, laboratory findings, clinical outcome, and the detection rates of viral pathogens between non-ICS group and ICS group

Characteristics	Non-ICS group n=26	ICS group n=28	p value
Age, years[a]	9.2±2.2	10.3±2.5	0.086
Men	17 (65.4%)	14 (50.0%)	0.25
Treatment during a stable state, n (%)			
LABA	0 (0.0%)	22 (78.6%)	< 0.001*
Symptom			
Wheezes	24 (92.3%)	22 (78.6%)	0.25
Duration of URTI symptoms before consultation, days[b]	4 (2–6)	3 (2–6)	0.83
Body temperature, °C[a]	37.3±0.6	37.3±1.0	1.00
Laboratory findings			
CRP, mg/dL[b]	0.49 (0.29–1.20)	0.41 (0.04–0.99)	0.18
WBC count, /μL[a]	9,337±2,588	7,603±2,700	0.02*
Eosinophil count in bloods, /μL[b]	66 (8–518)	312 (45–570)	0.14
Eosinophil percentage in blood, %[b]	0.8 (0.1–4.4)	3.6 (0.8–7.1)	0.07
Total serum IgE, IU/mL[b]	1,187 (742–1,891)	810 (435–1,746)	0.31
Hospitalization duration, day[b]	6 (5–8)	8 (7–8)	0.07
Treatment with systemic corticosteroids, n (%)	26 (100.0%)	27 (96.4%)	1.00
Systemic steroid treatment duration, days[b]	5 (4–7)	5 (4–7)	0.83
Viral pathogen detected	23 (88.5%)	22 (78.6%)	0.47
HRV/enterovirus[C]	21 (80.8%)	15 (53.6%)	0.03
HRV	21 (80.8%)	12 (42.9%)	0.004*
HRV-A	9 (34.6%)	7 (25.0%)	0.44
HRV-B	0 (0.0%)	1 (3.6%)	1.00
HRV-C	12 (46.2%)	4 (14.3%)	0.01*
non detected	0 (0%)	2 (7.1%)	0.49
enterovirus	0 (0.0%)	1 (3.6%)	1.00
Parainfluenza virus	2 (7.7%)	3 (10.7%)	1.00
Respiratory syncytial virus	0 (0.0%)	3 (10.7%)	0.24
Influenza A virus	1 (3.8%)	1 (3.6%)	1.00
Human metapneumovirus	0 (0.0%)	1 (3.6%)	1.00
Coronavirus	0 (0.0%)	1 (3.6%)	1.00
Adenovirus	0 (0.0%)	1 (3.6%)	1.00

Data are the mean±standard deviation[a] and the median (interquartile range)[b]
[C]Detected by multiplex PCR. *$p < 0.05$
Number of participants with missing data were as follow: 2 for CRP; 1 for WBC count and eosinophil count and percentage in blood, 8 for total serum IgE.
Abbreviations;PFT, pulmonary function test; ICS, inhaled corticosteroids; LABA, long-acting beta2-agonist; URTI, upper respiratory tract infection; CRP, C-reactive protein; WBC, white blood cell; IgE, immunoglobulin E; FVC, forced vital capacity; FEV1, forced expiratory volume in one second; HRV, human rhinovirus.
