# Supplementary material for: Effects of treatment with corticosteroids on human rhinovirus-induced asthma exacerbations in pediatric inpatients: a prospective observational study
Source: BMC Pulm Med. 2023 Dec 5;23:487. doi: 10.1186/s12890-023-02798-6 (PMC10696820; doi:10.1186/s12890-023-02798-6)
Supplement: Supplementary file 2 — Additional file 2. [file 12890_2023_2798_MOESM2_ESM.pdf]

Additional file 2. Comparison of characteristics, laboratory findings, and clinical outcome between non-PFT group and PFT group in HRV-positive hospitalized patients with systemic steroids treatment

Characteristics	Non-PFT group n=15	PFT group n=18	p value
Age, years[a]	9.4±1.9	10.4±2.6	0.19
Male, n (%)	10 (66.7%)	11 (61.1%)	0.74
Treatment during a stable state, n (%)			
ICS	0 (0.0%)	2 (11.1%)	0.49
ICS/LABA	0 (0.0%)	10 (55.6%)	< 0.001*
Anti-lgE antibody	0 (0.0%)	1 (5.6%)	1.00
Symptom			
Wheezes, n (%)	15 (100.0%)	14 (77.8%)	0.11
Duration of URTI symptoms before consultation, day[b]	3 (2–4)	4 (2–7)	0.74
Body temperature, °C[a]	37.2±0.5	37.3±0.9	0.56
Laboratory findings			
CRP, mg/dL[b]	0.60 (0.34–1.30)	0.42 (0.15–1.04)	0.26
WBC count, /µL[a]	9,373±2,341	8,549±2,667	0.35
Eosinophil count in bloods, /µL[b]	130 (8–592)	302 (68–507)	0.65
Eosinophil percentage in blood, %[b]	0.9 (0.1–6.2)	3.3 (1.2–6.4)	0.56
Total serum IgE, IU/mL[b]	1,351 (686–2354)	828 (516–1848)	0.28
Baseline lung function (pre-bronchodilator)			
FVC, L[a]	-	2.11±0.54	-
FEV1, L[a]	-	1.85±0.53	-
FEV1% predicted, %[a]	-	92.5±10.5	-
FEV1/FVC, %[a]	-	87.6±7.3	-
Types of human rhinovirus, n (%)			
A	5 (33.3%)	11 (61.1%)	0.11
B	0 (0.0%)	1 (5.6%)	1.00
C	10 (66.7%)	6 (33.3%)	0.06
Hospitalization duration, days [b]	6 (5–8)	7 (6–8)	0.19
Systemic steroid treatment duration, days [b]	5 (4–7)	6 (4–7)	0.85

Data are the mean±standard deviation[a] and the median (interquartile range)[b]

Number of participants with missing data were as follows; 2 for Total serum IgE.
*$p < 0.05$.

Abbreviations;PFT, pulmonary function test; ICS, inhaled corticosteroids; LABA, long-acting beta2-agonist; URTI, upper respiratory tract infection; CRP, C-reactive protein; WBC, white blood cell; IgE, immunoglobulin E; FVC, forced vital capacity; FEV1, forced expiratory volume in one second.
