# Supplementary material for: Effects of treatment with corticosteroids on human rhinovirus-induced asthma exacerbations in pediatric inpatients: a prospective observational study
Source: BMC Pulm Med. 2023 Dec 5;23:487. doi: 10.1186/s12890-023-02798-6 (PMC10696820; doi:10.1186/s12890-023-02798-6)
Supplement: Supplementary file 3 — Additional file 3. [file 12890_2023_2798_MOESM3_ESM.pdf]

Additional file 3. Baseline characteristics and laboratory findings by the treatment period of systemic steroids in HRV-positive hospitalized patients with followed-up PFT

Characteristics	Overall n=18	Steroid treatment period<br>3–5 days n=8	 6–8 days n=10	p-value
Age, years[a]	10.4±2.6	10.4±3.0	10.5±2.4	0.92
Men, n (%)	11 (61.1%)	3 (37.5%)	8 (80.0%)	0.14
Treatment during a stable state, n (%)				
ICS	2 (11.1%)	1 (12.5%)	1 (10.0%)	1.00
ICS/LABA	10 (55.6%)	4 (50.0%)	6 (60.0%)	1.00
Symptom				
Wheezes, n (%)	14 (77.8%)	5 (62.5%)	9 (90.0%)	0.27
Duration of URTI symptoms before consultation, days[b]	4 (2–7)	4 (2–6)	4 (2–7)	0.96
Body temperature, °C[a]	37.3±0.9	37.1±1.1	37.4±0.7	0.54
Laboratory findings				
CRP, mg/dL[a]	0.42 (0.15–1.04)	0.28 (0.07–0.69)	0.74 (0.24–1.04)	0.53
WBC count, /μL[a]	8,549±2,667	8,492±2,892	8,594±2,632	0.94
Eosinophil count in bloods, /μL[b]	302 (68–507)	251 (54–455)	312 (93–684)	0.62
Eosinophil percentage in blood, %[b]	3.3 (1.2–6.4)	3.3 (0.9–4.7)	3.3 (1.4–8.9)	0.76
Total serum IgE, IU/mL[b]	828 (516–1,848)	842 (321–1,544)	816 (575–1,913)	0.68
Baseline lung function (pre-bronchodilator)				
FVC, L[a]	2.11±0.54	2.16±0.52	2.08±0.58	0.76
FEV1, L[a]	1.85±0.53	1.96±0.55	1.77±0.52	0.45
FEV1% predicted, %[a]	92.5±10.5	97.7±9.3	88.2±9.8	0.053
FEV1/FVC, %[a]	87.6±7.3	90.3±6.5	85.3±7.4	0.15
Hospitalization duration, days[b]	7 (6–8)	6 (6–8)	8 (7–9)	0.17
Rhinovirus type, n (%)				
A	11 (61.1%)	5 (62.5%)	6 (60.0%)	1.00
B	1 (5.6%)	0 (0.0%)	1 (10.0%)	1.00
C	6 (33.3%)	3 (37.5%)	3 (30.0%)	1.00

Data are the mean±standard deviation[a] and the median (interquartile range)[b]

Statistical analysis was used with t-test or Mann-Whitney U test for continuous variables, and chi-squared test or Fisher's exact test for dichotomous variable between systemic steroid treatment duration for 3–5 or 6–8 days.

Two were missing for total serum IgE.

Abbreviations; PFT, pulmonary function test; ICS, inhaled corticosteroids; LABA, long-acting beta2-agonist; URTI, upper respiratory tract infection; CRP, C-reactive protein; WBC, white blood cell; IgE, immunoglobulin E; FVC, forced vital capacity; FEV1, forced expiratory volume in one second.
