# Supplementary material for: Effects of treatment with corticosteroids on human rhinovirus-induced asthma exacerbations in pediatric inpatients: a prospective observational study
Source: BMC Pulm Med. 2023 Dec 5;23:487. doi: 10.1186/s12890-023-02798-6 (PMC10696820; doi:10.1186/s12890-023-02798-6)
Supplement: Supplementary file 4 — Additional file 4. [file 12890_2023_2798_MOESM4_ESM.pdf]

Additional file 4. Comparison of changes in FEV_1% predicted from baseline in HRV-A-positive and HRV-C-positive patients.

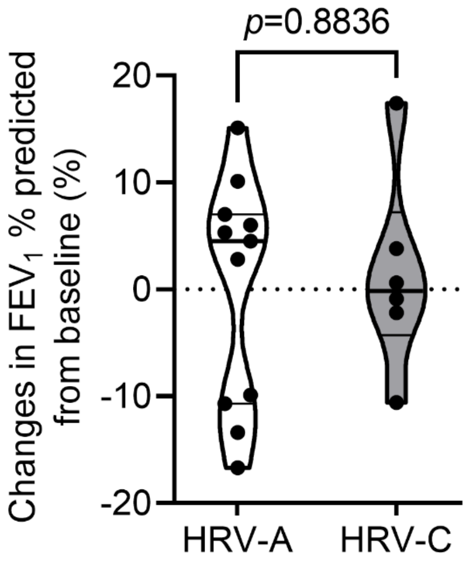

Eleven HRV-A-positive and 6 HRV-C-positive patients underwent a PFT 3 months after exacerbations, and FEV_1% predicted from baseline was compared. Values are expressed as the median with an interquartile range. Differences in data were analysed by the Mann–Whitney *U*-test. FEV_1, forced expiratory volume in one second.
